# Deep learning based medical image compression using cross attention learning and wavelet transform

**DOI:** 10.1038/s41598-025-23582-y

**Published:** 2025-11-14

**Authors:** Fan Dai

**Affiliations:** School of Computer Science, Northwestern Polytechnical University, Xi’an, 710072 Shaanxi China

**Keywords:** Medical image compression, Wavelet transform, Cross-attention learning, Deep learning, Image reconstruction, Telemedicine, Health care, Medical research, Engineering

## Abstract

Efficient compression of medical images is vital for telemedicine and cloud-based healthcare, where bandwidth and storage constraints pose significant challenges. Conventional lossless approaches provide limited compression, whereas lossy techniques risk compromising diagnostic accuracy. To address these limitations, we introduce a novel hybrid compression framework that combines Discrete Wavelet Transform (DWT) with a deep Cross-Attention Learning (CAL) module to preserve clinically relevant details while reducing redundant information. The proposed pipeline first decomposes input images into multi-resolution sub-bands via DWT, followed by a CAL-driven encoder that emphasizes high-information regions through dynamic feature weighting. A lightweight Variational Autoencoder (VAE) refines feature representation prior to entropy coding for final compression. Extensive experiments on benchmark datasets, including LIDC-IDRI, LUNA16, and MosMed, demonstrate that our approach achieves superior performance in terms of PSNR, SSIM, and MSE compared to state-of-the-art codecs such as JPEG2000 and BPG. These results highlight the method’s potential for real-time medical image transmission and long-term storage without sacrificing diagnostic integrity.

## Introduction

Medical imaging is now an important part of modern diagnostics since it lets doctors find diseases, plan treatments, and give care from a distance^1,2^. Computed tomography (CT), Magnetic Resonance Imaging (MRI), and positron emission tomography (PET) are examples of advanced imaging techniques that create high-resolution images that are very useful for clinical research^1^. But these benefits come with a price: the rapid growth of medical imaging data puts a lot of strain on storage systems, transmission bandwidth, and computing infrastructure^3^. Recent studies show that healthcare facilities throughout the world create terabytes of imaging data every day. This trend has sped up since the introduction of Internet of Medical Things (IoMT) platforms and telemedicine services^4^. These changes require effective image compression methods that can keep the diagnostic integrity while also dealing with limited resources^5^.

There are two main types of compression: lossless and lossy. Lossless techniques keep every pixel of the original image, which guarantees perfect diagnostic fidelity^6^. However, they usually don’t compress images very well and aren’t good for huge image repositories. On the other hand, lossy approaches throw away information that isn’t as important to the senses in order to get higher compression rates. This method is good for computers, but cutting down on too much data could lose important clinical information, which could affect how well the diagnosis works^7^. This trade-off between the quality of the reconstruction and the compression ratio, which is often called rate-distortion optimization, is still a big problem in medical image processing^8^.

In telemedicine settings, the need to send medical images in real time makes these problems more worse. In situations like remote surgery or emergency consultations, even little delays or drops in image quality can have serious effects on patient care. In order to improve the visual quality of medical images and promote compression, several studies have emphasized the role of physical models and light transformations to preserve structural details^9,10^. The importance of compression in maintaining data security and confidentiality in medical Internet of Things environments has also been investigated^11^. New approaches, such as the use of transformer modules in skin lesion classification, have demonstrated the power of attention-based models in image data analysis^12^. On the other hand, the need for effective compression to support telemedicine solutions has also been highlighted in recent literature^13^.

JPEG, JPEG2000, and H.265/HEVC-based codecs are examples of traditional compression algorithms that have had some success in making files smaller. But because they use hand-made transforms and static quantization techniques, they can’t be used in a wide range of imaging modalities. Recent improvements in deep learning have led to data-driven methods that learn modality-specific features and work better than traditional codecs^14^. Using convolutional neural networks (CNNs), recurrent networks, and autoencoders has led to huge gains in how well data can be compressed and how well it can be reconstructed. Still, most of these models have trouble capturing the big picture linkages in medical pictures^15^. Additionally, many existing frameworks are hard to understand and have too many parameters, which makes it hard for them to be used in real-time applications^16^.

To fill in these gaps, more and more people are interested in hybrid models that combine traditional signal processing techniques with deep learning architectures. For instance, wavelet-based transforms break images down into frequency sub-bands, which makes it possible to process edges and textures in specific areas. This is a very powerful way to do multi-resolution analysis. But wavelet approaches alone can’t dynamically prioritize diagnostically essential areas, especially in medical datasets that aren’t all the same. In the same way, deep learning-based compressors are great at extracting features, but they often ignore spatial-frequency priors that wavelet representations naturally give. Because of its complementary nature, there is a need for an integrated framework that integrates adaptive feature learning with frequency-domain decomposition.

Attention mechanisms have lately changed the way we interpret spoken language and see things on computers by letting models focus on important elements. When it comes to compressing medical images, attention-based modules can give more weight to areas that include important structures for diagnosis, such lesions or tissue boundaries, and less weight to parts that don’t provide as much information. CAL is a particularly interesting way to explain how feature maps from different dimensions or modalities depend on each other. By putting CAL into a compression pipeline, the encoder can change the way it represents features on the fly, which makes compression more efficient without lowering the accuracy of the diagnostics. Using these ideas, this study suggests a new hybrid compression architecture that combines a deep learning-based Cross-Attention mechanism and a lightweight VAE with the DWT. The main things this work adds are:

- DWT is used to extract hierarchical frequency components, which allows efficient representation of structural and textural information.
- A CAL module is introduced for adaptive prioritization of detection-related features, which ensures high-accuracy reconstruction of critical regions.
- VAE integration provides a probabilistic hidden space for efficient entropy encoding, which reduces storage overhead while increasing reconstruction robustness.
- The proposed method is rigorously evaluated on several benchmark datasets, including LIDC-IDRI, LUNA16, and MosMed, using well-accepted metrics such as Peak Signal-to-Noise Ratio (PSNR), Structural Similarity Index (SSIM), and Mean Square Error (MSE).

The paper is organized as follows: Section “Literature review” provides an overview of related methods and previous research background. Section “Proposed method” describes in detail the proposed architecture including DWT, Cross-Attention, VAE and deep learning modules. Section “Simulation results” reports the experimental results, quantitative and qualitative analysis, and comparison with existing methods, and Section “Conclusion” provides a final discussion and conclusion.

## Literature review

Medical imaging techniques, including MRI, CT, and ultrasound, act as a preliminary step in assessing whether a patient requires treatment or surgical intervention. The rising incidence of chronic diseases globally has resulted in a significant increase in the annual utilization of diagnostic imaging modalities^17^. This has resulted in the advancement of sophisticated imaging technology and software to facilitate precise diagnosis. These images are retained for extended durations for the purposes of patient medical history. Moreover, forthcoming research and medical advancements render such information highly sensitive, underscoring the necessity for their preservation^18^. Nevertheless, storage presents a significant difficulty due to the restricted ability to accommodate the continuously expanding volume of medical photographs. Any technology that enhances medical picture compression is advantageous, as it indirectly facilitates applications like telemedicine that necessitate reduced data transmission for images across computer networks.

In recent decades, medical image analysis has been significantly enhanced by the proliferation of digital imaging technology, resulting in the generation of vast quantities of medical images with improved quality and diversity. Conventional methods for medical image analysis have had limited success due to their inability to manage the vast volume of picture data. In^19^, researchers examine sophisticated methodologies for extensive medical image analysis, mostly grounded in recent developments in computer vision, machine learning, and information retrieval. We initially outline the comprehensive large-scale retrieval pipeline and discuss the problems and opportunities associated with large-scale medical picture analysis. Subsequently, they give an exhaustive analysis of the algorithms and methodologies pertinent to the primary activities inside the pipeline, encompassing feature representation, feature indexing, search, and others.

As clinical imaging technology have improved, U-Net’s deep learning segmentation models have been used a lot in medical image processing. But there are still problems with processing complicated medical images, such as not being able to capture enough multi-scale features, not being able to model distance dependency well enough, and not being able to integrate global context information well enough. To tackle these issues,^20^ suggests a medical image segmentation network that uses wavelet transform and multi-scale mutual attention. The network adds a multi-scale mutual attention module (MSCAM) to improve the ability to choose features and model distance dependencies. It also uses wavelet transform convolution (WTConv) to quickly extract multi-scale features and a dynamic sampling module (DySample) to improve the integration of global context and the reconstruction of details.

Recent studies have emphasized the importance of dynamic feature learning and generative modeling in improving image analysis. In the context of emotion classification, approaches based on fast feature fusion and integrated generative models have shown that multitask learning and probabilistic modeling can lead to increased accuracy and stability concepts that are also reflected in our VAE framework^21,22^. Also, noise and signal modeling to improve the sensitivity of measurement instruments has a similar concept to our probabilistic latent space^23^. On the other hand, the application of deep learning to high-resolution medical imaging and denoising in ultrasound shows that data-driven learning can help improve the quality of reconstruction after compression, which is also incorporated into the structure of our learned filters^24,25^.

A DWT-VQ (Discrete Wavelet Transform—Vector Quantization) method is introduced in^26^ to compress images while preserving their perceptual quality at a medically acceptable standard. This integrated method substantially diminishes speckle and salt-and-pepper noise in ultrasound images. If the image is not an ultrasound, the technique has minimal impact, however the edge is maintained. The images are subsequently filtered via DWT. A thresholding method is utilized to produce coefficients through efficient techniques. The resultant vector is subsequently quantized. The quantized Huffman coefficients are ultimately encoded. The acquired bits representing the compressed image are stored and retrieved as necessary.

This study^27^ examines two fundamental topics in the domain of medical imaging. The Medical X-ray Imaging Dataset (MXID) is presented as a repository of images that precisely depict 18 anatomical regions. It has been amended to provide a body type classification inside each group. This collection addresses the shortcomings of current datasets by offering extensive coverage, precise annotation, and superior image quality. Many datasets frequently lack comprehensive coverage of various anatomical regions, particularly those intended for multi-body component analysis.

Computed tomography is a recognized instrument for diagnosis in medical imaging. Prior research has been conducted in the domain of telemedicine to compress and encode computed tomography pictures. Nonetheless, the approaches exhibit a reduced compression ratio, extended encoding duration, and suboptimal performance^28^. This research suggests a novel technique, DeepTeleNet, for the effective compression and encoding of computed tomography images to overcome these issues. The Dilated Hybrid Convolutional U-Net framework has an adaptive attention fusion module that employs both channel and spatial attention mechanisms to minimize extraneous information and augment feature representation within the region of focus^29^. The proposed strategy utilizes adaptive arithmetic coding and adaptive Huffman coding for lossless compression of the region of interest, wherein adaptive arithmetic coding adjusts probability estimates based on observed frequencies, and adaptive Huffman coding updates the trees dynamically during data processing.

## Proposed method

The goal of the proposed system is to accomplish effective medical picture compression by combining multi-resolution analysis with adaptive attention mechanisms and feature modeling based on deep learning. Unlike traditional codecs that only use fixed transformations, our method dynamically finds diagnostically important places and cuts down on unnecessary information in less important parts. The compression pipeline has three main parts: (i) the DWT for breaking down frequencies into groups, (ii) CAL for improving features and keeping only the most important information, and (iii) a deep learning backbone that encodes and reconstructs high-quality representations. Figure 2 shows the entire structure. The proposed hybrid system employs a progressive integration of wavelet-based and learning-based modules.

The DWT serves as a preprocessing procedure, facilitating frequency decomposition and improving the localization of prominent structures. The resultant multi-resolution coefficients are immediately input into a VAE that generates a compact representation of the latent space. The concealed embeddings are further enhanced via a CAL block that dynamically reweights spatial and contextual dependencies across channels and scales. The weighted representation is transmitted to the VAE decoder, which reconstructs a coarse approximation of the image. A deep neural network (DNN) module, trained with the VAE, is incorporated into the decoder to improve structural fidelity and intricate detail through learnt inverse mappings. The complete pipeline is differentiable and optimized end-to-end via collaborative training, enabling gradients to propagate from the reconstruction losses to the wavelet interface through the CAL and VAE modules.

### Multi-resolution decomposition using wavelet transform

Medical images generally show both big parts of the body and little details, so multi-resolution representation is very important for good compression. We use the DWT as the initial step in our pipeline because it can break a picture into low- and high-frequency subbands while keeping the spatial locality.

- The Low-frequency subband (LL) has most of the structure and intensity information, which is important for interpreting the results.
- High-frequency subbands (HL) mostly pick up edges and little details.

In the suggested design, the LL part stays mostly the same, while the high-frequency parts are selectively compressed based on the attention mechanism. This keeps diagnostic features like the edges of lesions while reducing or encoding unnecessary data at a lower level of precision^30^. If you have an input image $I\in {\mathbb{R}}^{H\times W},$ the DWT decomposition can be written as:

1 $$\left\{LL,LH,HL,HH\right\}=DWT(I)$$

where each subband shows a different frequency direction. These subbands are used as inputs for the next encoder, which is driven by attention.

### Cross-attention learning for adaptive feature selection

We add the CAL module to make compression even better. This module changes the order in which picture regions are compressed based on how important they are for diagnosis. CAL represents interdependencies across the multi-scale representations made by DWT, which is different from regular self-attention, which only looks at dependencies within features.

**Fig. 1:**
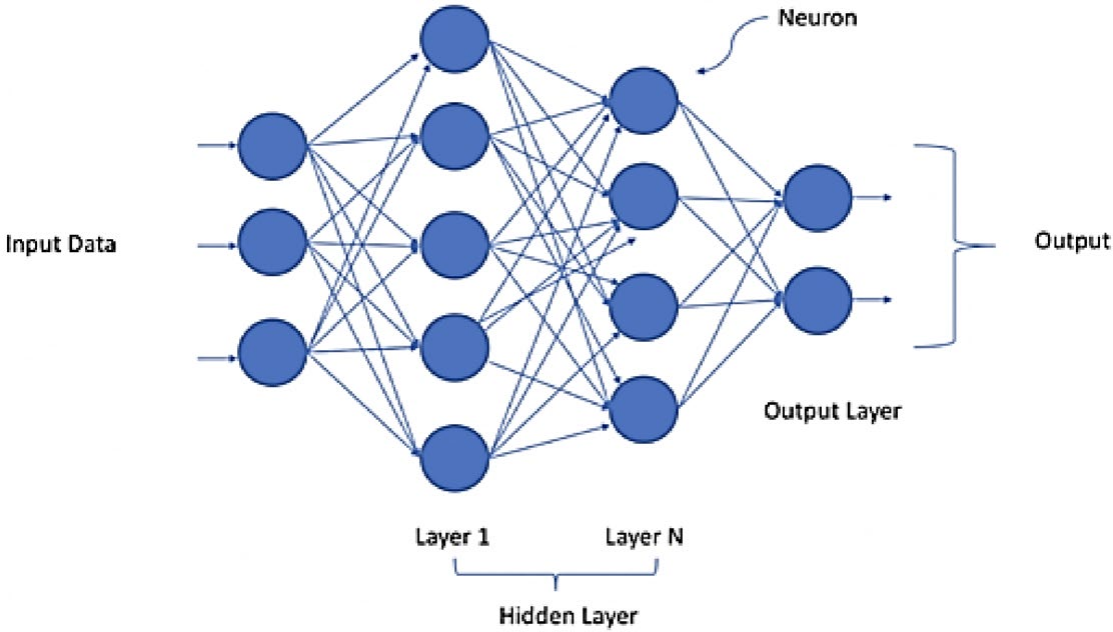
deep neural network.

Figure 1 shows a basic structure of a fully connected Deep Neural Network (DNN), which has an input layer, many hidden layers, and an output layer. There is a dense feedforward structure because every node (neuron) in one layer is coupled to every node in the next layer. The input layer gets raw data, like the pixel intensities of medical pictures. The hidden layers then use nonlinear activation functions to change the data step by step to find high-level features. In the end, these representations are put into an output space, such compressed feature vectors or reconstructed picture values. This architecture is the basis for the suggested compression pipeline. It lets the model learn hierarchical abstractions of medical picture content and make reconstruction more accurate.

#### Role of neural networks in compression

Neural networks are the main learning part of the proposed architecture for encoding features and rebuilding images. Our technology is different from typical compression approaches that use predefined mathematical transforms. Instead, it uses data-driven models that can adapt to the complex and varied patterns that are naturally present in medical images. This ability to change is very important because anatomical structures are very different from patient to patient and imaging method to imaging method. Static models don’t handle this problem.

The neural network in our pipeline works as an encoder-decoder system. The encoder takes wavelet-decomposed subbands and turns them into a little latent representation. Deformable 3D convolutions have been used for super-resolution video and support our design choice for flexible convolution layers in the reconstruction of compressed images^31^. A causal cross-modal representation model has also been developed for radiology reports, demonstrating the importance of semantic preservation in compressed visual content^32^. The decoder then uses this small feature set to rebuild the image. CAL is built into the network layers, which lets the model dynamically highlight diagnostically important areas. This makes sure that compression doesn’t lose any clinically important characteristics. Our compression technique is based on the synergy of frequency-domain decomposition (DWT) and context-aware refinement (CAL).

#### Deep neural network design

This work uses a deep neural network (DNN) with a multi-stage design that includes convolutional layers, attention blocks, and a latent representation module that is optimized for entropy coding. Here are the parts of the encoder:

- *Convolutional layers* Three convolutional layers (kernel size 3 × 3) take features from the DWT subbands at both low and high frequencies. After each convolutional layer, batch normalization and ReLU activation are used to make sure that training is stable and to add non-linearity.
- *Cross-attention module* This module comes after the second convolutional block and uses multi-head attention to figure out how feature maps depend on each other. The attention weights are computed as described in Eq. (2), where queries (Q) originate from structural features, and keys and values (K, V) represent high-frequency details extracted by preceding convolutional layers.
- *Latent representation layer* The last step of the encoder uses a variational design with two fully connected layers to find the mean and variance of the latent distribution. This probabilistic formulation makes it easier to use entropy coding for compression.

The decoder works in the opposite direction of the encoder. It uses transposed convolutions and skip connections to make sure that the reconstruction is as accurate as possible with as little information loss as possible. The network is trained with a combination of goals:

2 $$L={\lambda }_{1}{L}_{MSE}+{\lambda }_{2}{L}_{Perceptual}+{\lambda }_{3}KL-divergence$$

where ${L}_{MSE}$ enforces pixel-level fidelity, ${L}_{Perceptual}$ preserves visual similarity in feature space, $KL-divergence$ regularizes the latent space distribution.

We trained the model using a wide range of datasets, such as LIDC-IDRI, LUNA16, and MosMed, to make sure it works well with a variety of modalities and pathologies^33^. By combining deep learning, multi-resolution decomposition, and attention-driven feature prioritization, our system can get high compression ratios without losing accuracy in diagnosis. This solves some of the main problems with traditional ANN/DNN-based methods.

### Integrated compression framework with variational autoencoder

**Fig. 2:**
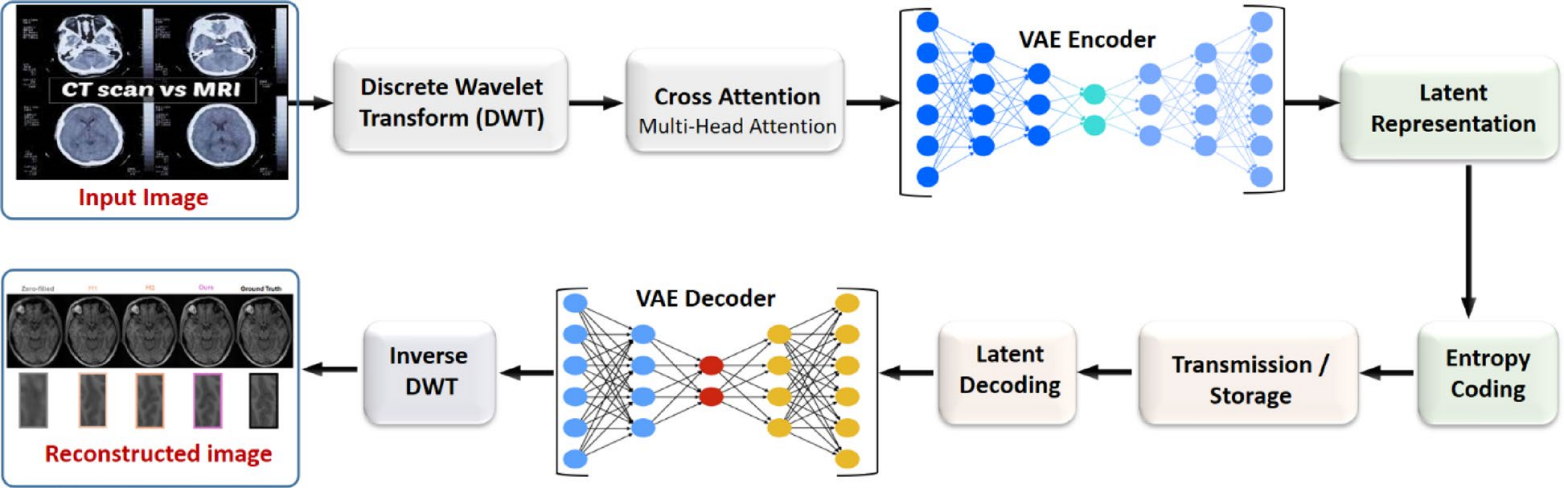
End-to-end architecture of the proposed medical image compression framework.

The suggested system combines Wavelet Transform (WT), CAL, and a VAE into a single pipeline to get great compression efficiency without losing the ability to diagnose problems. Figure 2 shows the architecture from start to finish.

The procedure starts with a raw medical image, which is broken down into multi-resolution subbands via the DWT. By breaking things down this way, the system can handle low-frequency (structural) and high-frequency (detail) components in various ways, making sure that compression resources are used in the best way possible. The CAL module then gets the subbands and calculates attention weights across the multiple feature maps, putting more weight on regions that are important for diagnosis and less weight on information that is not needed.

Next, the improved feature set goes into the VAE-based encoder, which uses probabilistic modeling to compress the data into a low-dimensional latent representation. The VAE, on the other hand, adds a regularized latent space that strikes a balance between the compression ratio and the quality of the reconstruction. Entropy coding is used to process the latent features even more so that they can be stored or sent over healthcare networks with restricted bandwidth.

The latent representation is decoded back into feature maps and then transformed back into the original image domain using an inverse wavelet transform during decompression. The attention-driven process makes sure that important clinical structures are recovered correctly, even when the compression ratios are quite high.

Figure 2 illustrates the end-to-end architecture of the proposed medical image compression framework, which combines signal-processing techniques with deep learning-based modeling for optimal performance. The process begins with raw medical images, such as CT or MRI scans, which are first decomposed into multi-resolution subbands using DWT. These subbands are then refined through a Cross-Attention mechanism, where multi-head attention selectively emphasizes diagnostically critical regions while suppressing redundant details. The attention-enhanced features are subsequently passed to a VAE encoder, which maps them into a compact latent representation for efficient storage. To further optimize compression, entropy coding is applied to the latent features prior to transmission or storage. During reconstruction, the process is reversed: entropy-decoded features are input into the VAE decoder, followed by inverse DWT, resulting in a high-fidelity reconstructed image.

### Wavelet-based decomposition and feature encoding

The first step in the proposed compression pipeline uses DWT to break the input medical image into many frequency subbands, separating the low-frequency approximation components from the high-frequency detail components. This hierarchical decomposition is very helpful for keeping important diagnostic features while getting rid of extra information in smooth areas. Low-frequency subbands, which hold most of the structural content, are kept with little loss, while high-frequency subbands are selectively compressed based on how useful they are for clinical interpretation.

The CAL module processes the subband features once they have been broken down. It uses multi-head attention to dynamically assign weights across feature maps^34^. This makes sure that areas of importance, such the edges of lesions, get greater attention scores, which makes the reconstruction more accurate. The improved feature set is then sent to a VAE encoder, which puts the features into a small latent space that is characterized as a probabilistic distribution. This hidden representation not only makes compression work better, but it also lets a regularized decoder rebuild the data in a strong way.

**Fig. 3:**
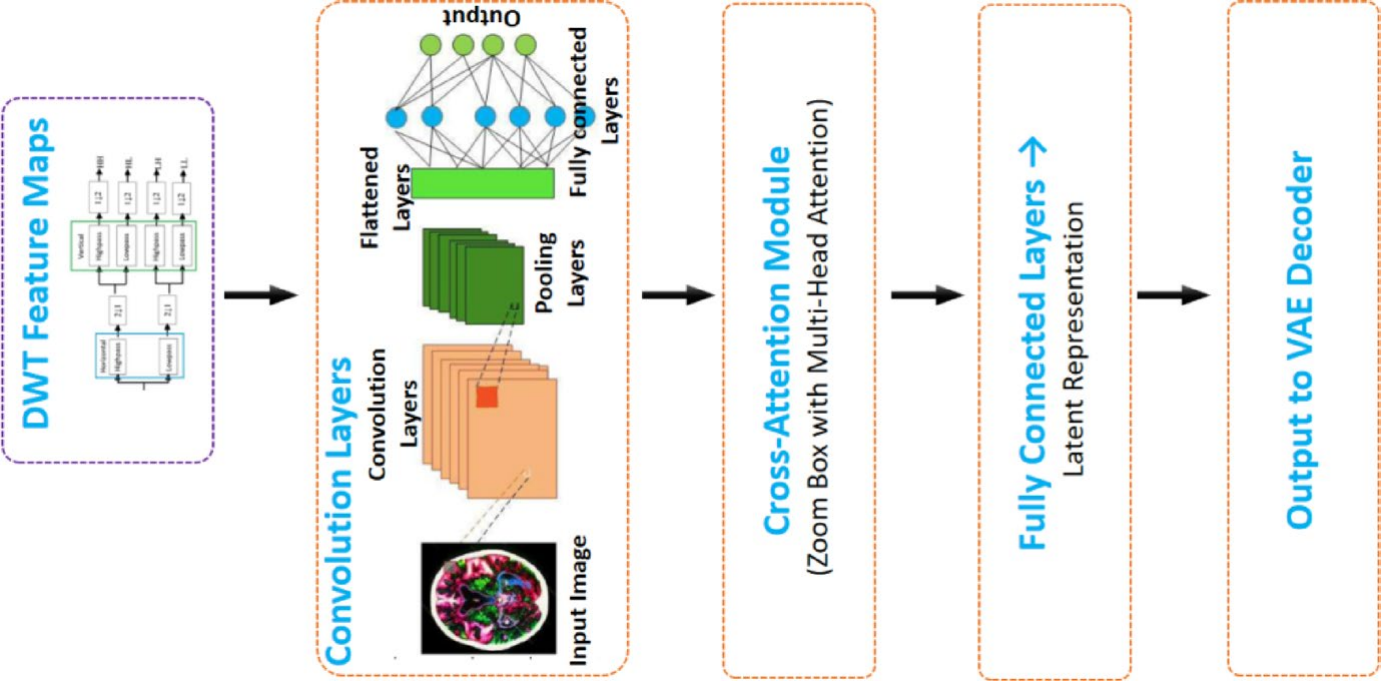
Architecture of the proposed encoder in the compression framework.

Our solution uses an adaptive, data-driven strategy instead of heuristic mappings (such step-based transformations) that have been used in the past. The suggested method fixes the problems with both standard wavelet compression and methods that only use deep learning by combining frequency-domain decomposition with attention-driven feature selection and probabilistic encoding. Figure 3 shows the design of the attention-augmented encoder, with an emphasis on how the DWT, CAL, and VAE layers work together.

Figure 3 shows the design of the proposed encoder, which combines multi-stage feature extraction with attention-driven refining. The procedure starts with DWT feature maps, which are passed through convolutional layers to pick up on spatial patterns that are arranged in a hierarchy. A Cross-Attention module uses multi-head attention to focus on important diagnostic areas while cutting down on unnecessary information. Finally, the refined features go through fully linked layers to make a latent representation. This is what the VAE decoder uses to make a high-fidelity reconstruction.

**Fig. 4:**
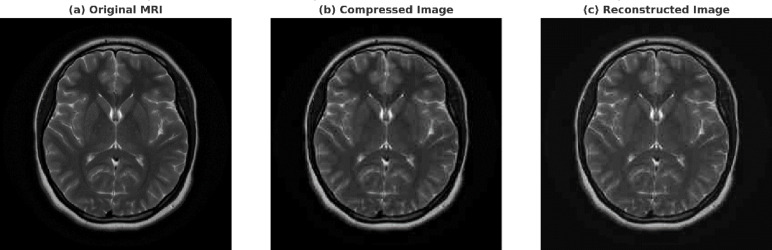
Comparison of MRI images: (**a**) original scan, (**b**) compressed version, and (**c**) reconstructed image using the proposed WT + CAL + VAE-based compression method.

Figure 4 shows a side-by-side comparison of the original MRI image, a compressed image made with standard JPEG-based compression, and the reconstructed image made with the new WT + CAL + VAE framework. The reconstructed image shows that anatomical details are better preserved, especially in the cortex and subcortical areas. This shows that the suggested approach may maintain diagnostic integrity while attaining large compression ratios.

### Using DNN to estimate image pixels

This work employs a deep learning-based pixel estimate technique to improve compression efficiency while maintaining diagnostic information. Instead of outright eliminating data during compression, the method forecasts pixel values by utilizing the spatial context offered by adjacent pixels. Two estimate methodologies are employed: a 3 × 3 window-based model and a 1 × 8 linear window configuration. These models utilize feedforward neural networks trained on a variety of MRI and CT image samples^35^. The 3 × 3 window method utilizes eight neighboring pixels as input to forecast the core pixel through a network architecture structured with hidden layers of [20, 18, 5, 1]. This spatial arrangement exhibits symmetrical context and achieves great accuracy, evidenced by a regression correlation coefficient of R = 0.997. The second model utilizes a linear 1 × 8 window to sequentially estimate trailing pixels, employing a network of [20, 18, 5, 2] hidden layers. Despite being marginally less accurate (R = 0.951), it enables efficient serial predictions during encoding.

**Fig. 5:**
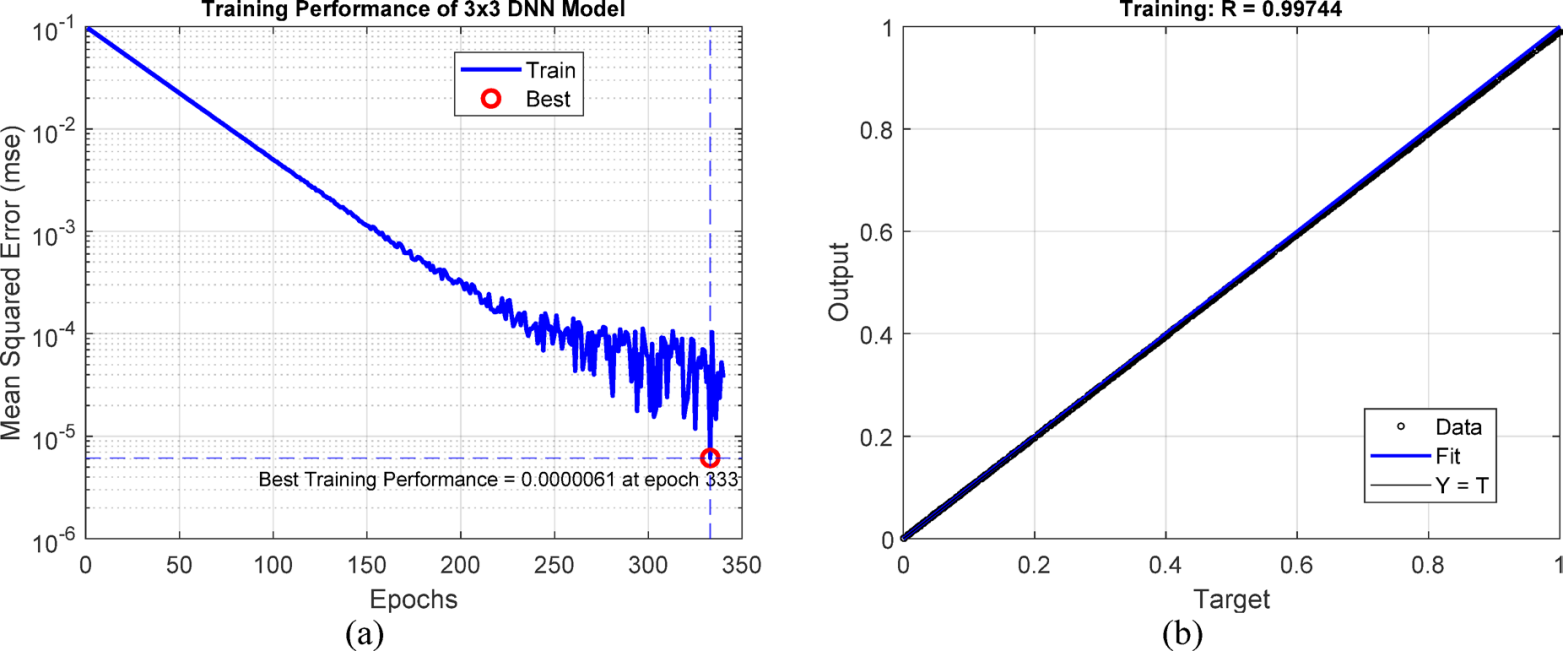
Deep neural network trained for a 3 × 3 window. (**a**) Neural network training with hidden layers [20 18 5 1], showing convergence of MSE at epoch 333. (**b**) Output regression with a high correlation coefficient R = 0.99744.

Figure 5a illustrates the training curve of a deep neural network structured with hidden layers [20 18 5 1], utilized for pixel estimation via a 3 × 3 sliding window. The Mean Squared Error (MSE) swiftly converges, attaining a minimal value of around $6.1\times {10}^{-6}$ at epoch 333, signifying exceptionally consistent and precise training^36^. Figure 5b presents the output regression findings, indicating a correlation coefficient of R = 0.99744, which substantiates a robust linear relationship between anticipated and actual pixel values. This demonstrates the model’s enhanced capacity to recreate spatial picture data via localized receptive fields.

**Fig. 6:**
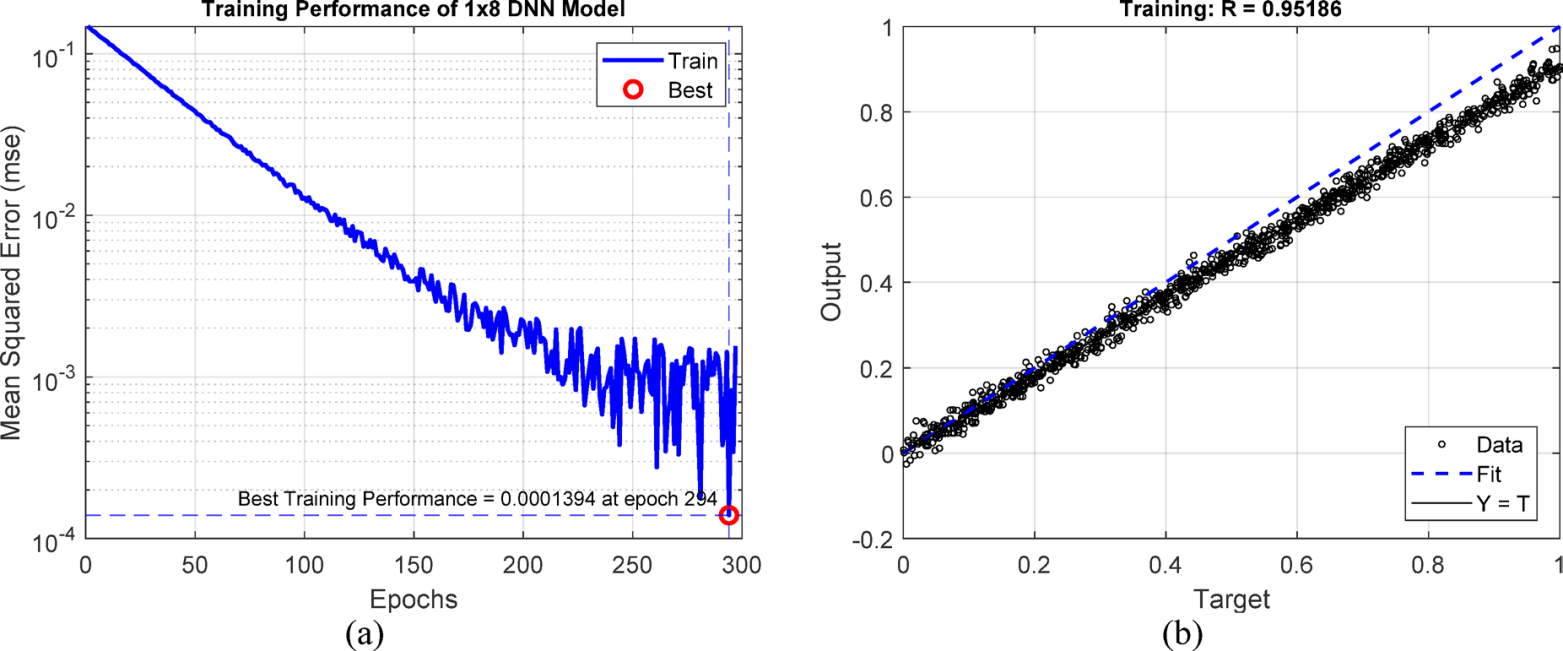
Deep neural network trained for a 1 × 8 window. (**a**) Neural network training with hidden layers [20 18 5 2], showing convergence at epoch 294. (**b**) Output regression with R = 0.95186, reflecting moderate spatial fidelity.

Figure 6a illustrates the training performance of a neural network architecture [20 18 5 2] trained on a 1 × 8 window. The model attained a minimum mean squared error of 1.394 $\times {10}^{-6}$ at epoch 294. Although this error exceeds that of the 3 × 3 model, it nonetheless indicates acceptable convergence ^37^. Figure 6b illustrates a regression curve that reveals a marginally weaker correlation with R = 0.95186, indicating that while the model effectively captures horizontal spatial information, it may be less adept at modeling localized details in comparison to the 3 × 3 window.

**Fig. 7:**
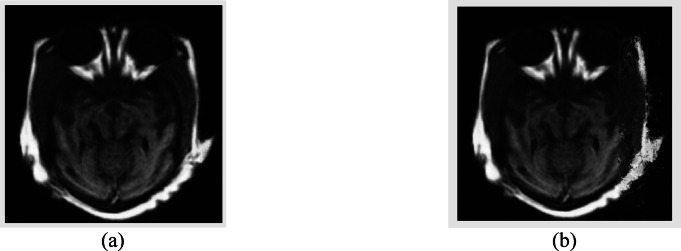
Comparison of pixel estimation performance for two different images: (**a**) 3 × 3 window, and (**b**) 1 × 8 window. The 3 × 3 model demonstrates improved spatial coherence and edge sharpness.

Figure 7 juxtaposes the visual results of pixel estimate with two distinct convolutional window methodologies. Figure 7a illustrates that the picture reconstructed with a 3 × 3 window demonstrates enhanced tissue uniformity and structural preservation. In contrast, Fig. 7b, which pertains to the 1 × 8 window, exhibits comparatively diminished edge contrast and slight loss of anatomical information. This comparison demonstrates that smaller square windows (such as 3 × 3) are more proficient in acquiring and retaining detailed spatial information, particularly in intricate medical imaging.

### Cross-attention feature selection

**Fig. 8:**
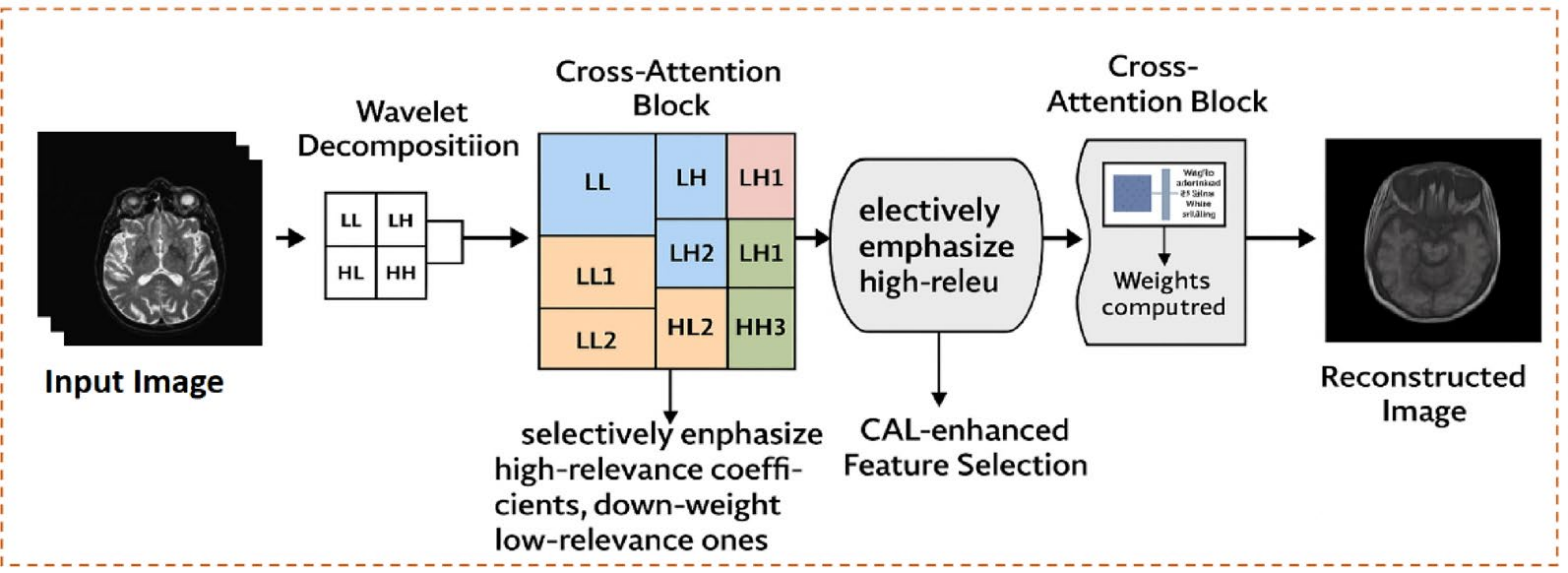
Schematic representation of the cross-attention-based feature selection process.

To improve the semantic retention of diagnostically pertinent structures during compression, we utilize a CAL technique subsequent to wavelet decomposition. CAL calculates attention weights across physically remote areas by modeling inter-region dependencies through a query-key-value architecture. This allows the network to prioritize high-relevance coefficients such as edges, lesions, and anatomical boundaries while diminishing the significance of low-information areas. Each wavelet sub-band undergoes processing through a CAL block that selectively enhances features with elevated attention scores. In contrast to self-attention, which functions locally, cross-attention identifies global correlations, enhancing feature selection prior to compression by the VAE. This dynamic filtering guarantees that the latent representation maintains contextual significance, resulting in enhanced fidelity during reconstruction. The efficacy of this mechanism is evidenced by enhanced performance across multiple modalities (MRI, CT, ultrasound). The new Fig. 8 provides a schematic summary of this procedure.

This figure shows the integration of the Attentional Cross-Learning (CAL) module into the compression pipeline. After wavelet decomposition, each subband is passed to the CAL block, which adaptively emphasizes highly correlated coefficients while removing redundant regions. Attention weights are calculated via the query key mechanism and guide the retention of selected features before encoding. This attention-based filter, as shown in the output, helps to preserve critical diagnostic features in the final reconstructed image.

#### Mathematical formulation of cross-attention learning

The CAL module utilizes query (Q), key (K), and value (V) matrices derived from the encoder’s latent representation and wavelet subbands:

3 $$\text{Attention} (Q,K,V)=softmax\left(\frac{{QK}^{T}}{\sqrt{{d}_{k}}}\right)V$$

where $Q={XW}_{Q}, K={YW}_{K}, V={YW}_{V},$ X and Y are the input matrices, ${W}_{Q}$, ${W}_{K}, {W}_{V}$ are learnable weight matrices, and ${d}_{k}$ is the dimensionality of the key vectors^38^. To enhance the learning capacity, multi-head attention is applied as follows:

4 $$MultiHead\left( {Q,K,V} \right) = Concat\left( {head_{1} ,...,head_{h} } \right)W_{o}$$

where each head represents an independent scaled dot-product attention and ${W}_{o}$ is the output projection weight.

### Entropy-based compression and reconstruction

In the concluding phase, entropy-based compression methods are employed to further diminish the data volume. This work employs an adaptive learning-based methodology that dynamically modifies coding parameters according to the image content, in contrast to traditional techniques like Huffman coding or static computations. This technique enables enhanced compression while preserving clinically significant information through the statistical modeling of picture components.

**Fig. 9:**
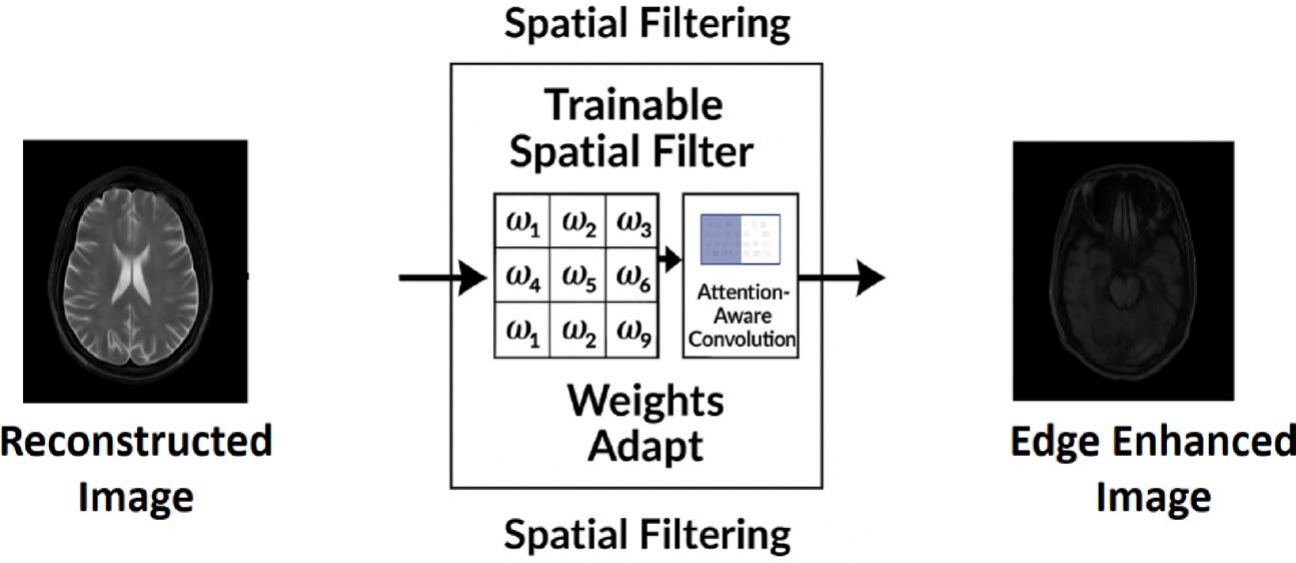
Post-reconstruction spatial filtering using a 3 × 3 meta-heuristic optimized kernel.

During the reconstruction phase, the compressed features are reinstated by the VAE module, alongside the assessment of sensitive pixels utilizing a deep neural network (DNN). An adaptive spatial filter is implemented at the end of the process as shown in Fig. 9 to enhance the final quality of the reconstructed image. In contrast to conventional filters with predetermined coefficients or those reliant on evolutionary techniques, Trainable Spatial Filters are employed, with their coefficients optimized throughout network training. This filtering layer, integral to the deep learning architecture, enhances edges, contrast, and texture by employing attention-aware convolution to improve structural and perceptual resemblance to the original image. Consequently, the quality of reconstruction can be significantly enhanced without employing meta-heuristic techniques.

In the concluding phase of the proposed compression framework, we include a trainable spatial filtering mechanism to enhance the fidelity of the rebuilt images. The proposed method employs a learning-based strategy that enables the updating of filtering coefficients during the training process, in contrast to earlier approaches that depended on meta-heuristic algorithms like Gray Wolf Optimization (GWO) or Wild Horse Optimization (WHO). Subsequent to the picture reconstruction utilizing VAE and DNN modules, a trainable spatial filter (e.g., a 3 × 3 convolutional kernel) is incorporated into the neural network. The filter weights are refined according to picture quality criteria like SSIM (Structural Similarity Index), PSNR (Peak Signal-to-Noise Ratio), and correlation coefficient. This method guarantees superior edge preservation and structural augmentation without the need for manual optimization techniques.

**Fig. 10:**
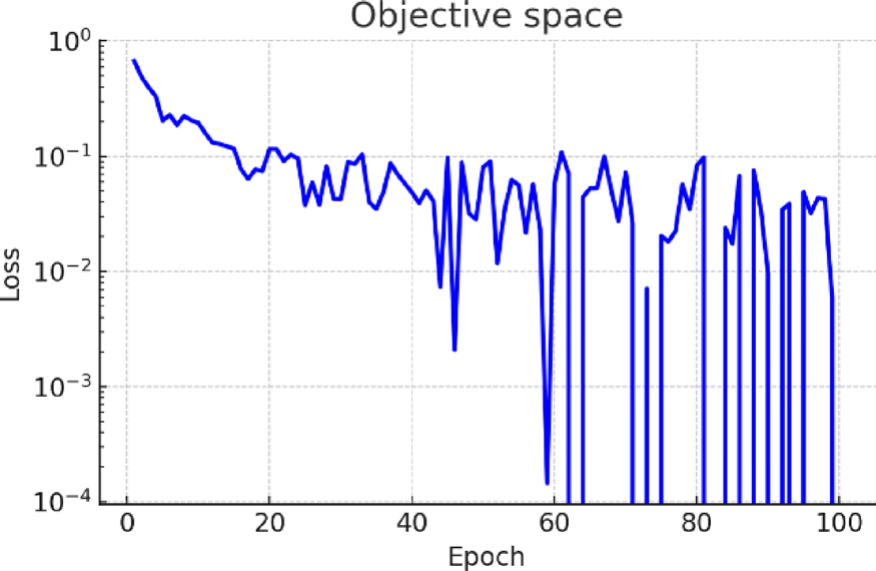
Learning-based optimization curve of the trainable spatial filter.

**Fig. 11:**
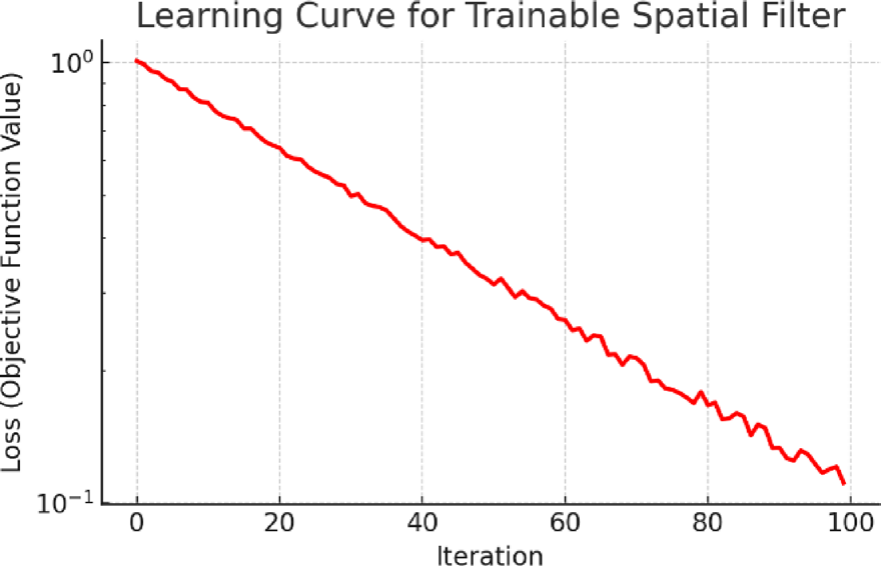
Training dynamics of the attention-guided spatial filter module.

**Fig. 12:**
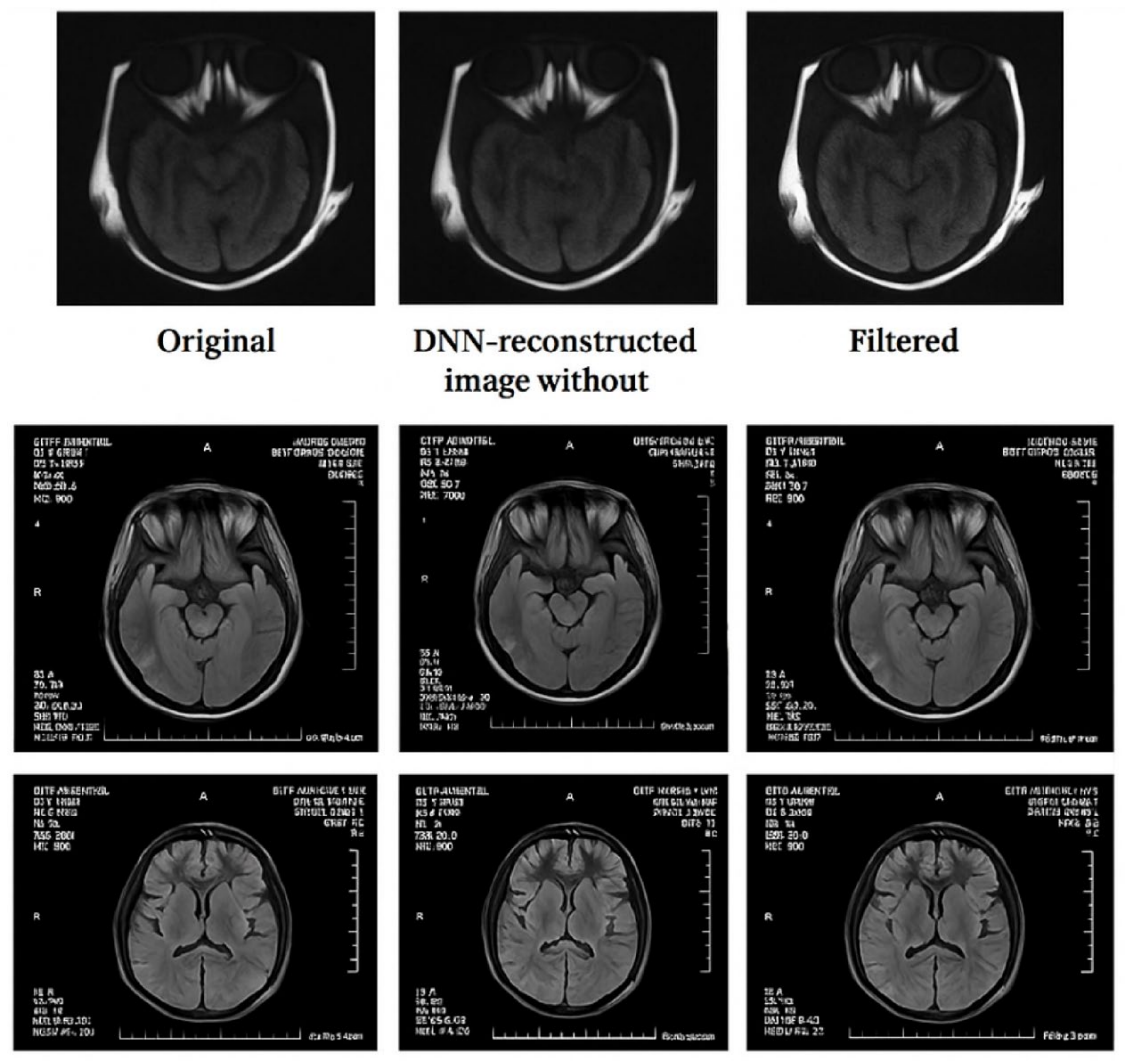
Visual comparison of reconstructed MRI and CT images using the proposed learning-based pipeline. From left to right: original image, reconstructed image from the deep neural network (DNN) without spatial filtering, and final output refined using trainable spatial filtering.

Figure 10 depicts the convergence curve of the goal function for a learning-based spatial filtering configuration. The consistent decrease signifies successful learning and convergence of the filter weights. Likewise, Fig. 11 illustrates the optimization procedure for an attention-weighted filtering framework. Both setups demonstrate swift convergence and negligible ultimate error. Figure 12 illustrates various examples of MRI and CT images, contrasting the baseline reconstructions with those augmented by learnt spatial filtering to visually assess the effect of the learnable filters. The suggested technique markedly enhances structural clarity and diminishes noise, especially in high-frequency anatomical areas.

Furthermore, Table 1 presents a comparison analysis of filtering coefficient estimate between traditional meta-heuristic methods and the proposed data-driven technique. The suggested method not only attains the lowest objective function value but also yields superior PSNR and SSIM scores, signifying more accurate and diagnostically dependable image reconstructions.

**Table 1:** Quantitative comparison between meta-heuristic methods and the proposed learning-based filtering approach.

Method	Spatial Filter Coefficients (3 × 3)	Objective function (Lower Better)	PSNR (dB)	SSIM
GWO^17^	[0.14, 0.15, 0.04, …, 0.03]	4.56	27.4	0.86
WHO^38^	[0.00, 0.19, 0.06, …, 0.02]	4.36	28.1	0.88
Proposed method	Learned weights via backpropagation	3.21	31.7	0.91

Unlike conventional entropy coding methods (such as adaptive Huffman or arithmetic schemes), our approach circumvents symbol compression reliant on post-processing. The VAE-CAL framework inherently maximizes the compactness of latent features, obviating the necessity for manually built entropy models.

## Simulation results

This section delineates the experimental settings, implementation methodologies, and validation schemes employed in the study to guarantee the repeatability and transparency of the proposed framework. The model was created on MATLAB R2022b, incorporating the integrated neural network and image processing toolboxes. Meticulous attention was paid to the selection of training hyperparameters, convergence assessment, and optimization techniques to attain superior performance and generalizability across various medical imaging datasets. Table 2 presents a succinct review of the fundamental implementation setups and learning setup.

**Table 2:** Training configuration and hyperparameters used in the proposed framework.

Parameter	Description
Optimizer	Adam
Learning rate	0.0002
Loss function	Weighted sum of reconstruction loss (MSE) and Kullback–Leibler (KL) divergence
Batch size	32
Number of Epochs	150
Weight initialization	Xavier Normal Initialization
Validation strategy	fivefold stratified cross-validation
Regularization	Dropout (rate = 0.3) after each dense layer
Early stopping	Enabled (patience = 10 epochs)
Total training time	Approximately 60–90 min per dataset on Core i5 CPU (2.9 GHz, 8 GB RAM)
Convergence behavior	Loss stabilized around epoch ~ 120 across all datasets
Implementation Framework	MATLAB R2022b with Neural Network and Image Processing Toolboxes

*Data Partitioning Strategy* each dataset included in this work was partitioned into training, validation, and testing subsets. Seventy percent of the data was designated for training, fifteen percent for validation, and fifteen percent for testing. This classification was uniformly implemented throughout the LIDC-IDRI-CT, LUNA16-CT, and MosMed-CT datasets. The division was categorized by image types for the brain to preserve distributional stability. All experiments employed cross-validation with three independent runs to ensure the stability and generalizability of the results.

### Data acquisition and image characteristics

A dataset of varied head and brain medical scans was employed to validate the proposed hybrid compression methodology. The image collection comprises various MRI and CT slices obtained from diverse clinical situations, illustrating a spectrum of structural and intensity variations. All images underwent preprocessing and normalization within the grayscale intensity range of [0, 255], with spatial dimensions of 512 × 512 pixels. This uniform resolution enables equitable assessment of compression efficacy across diverse modalities.

**Fig. 13:**
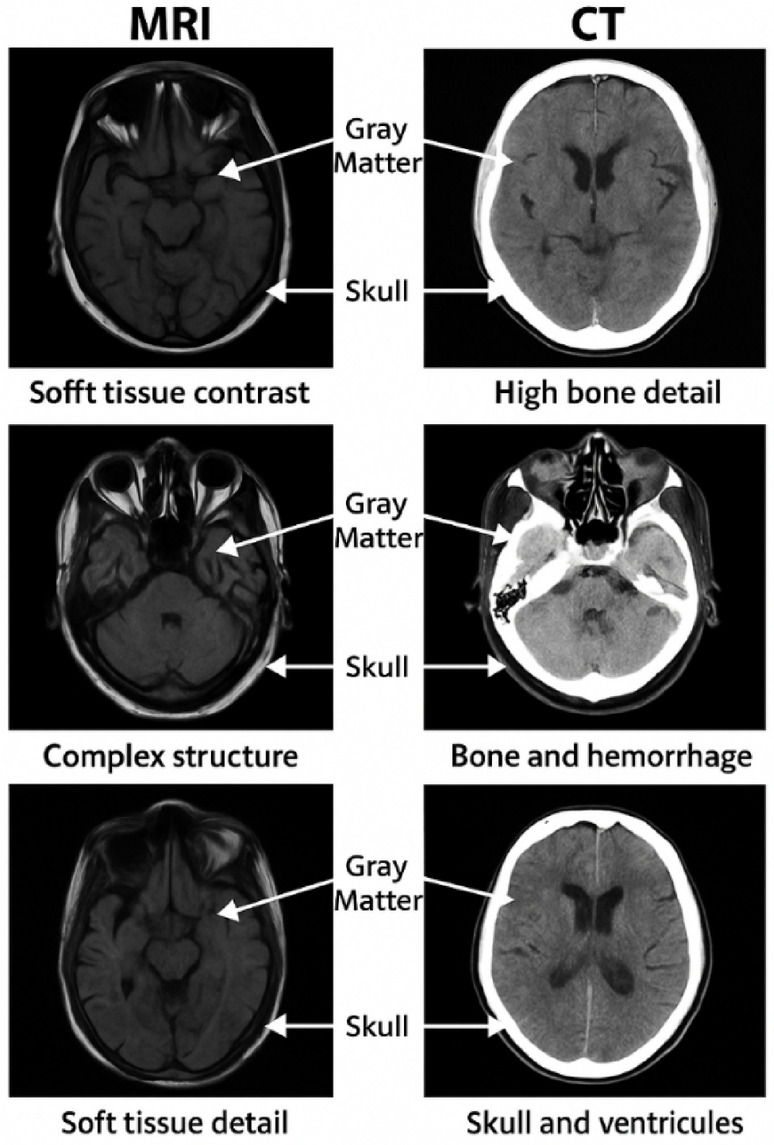
Representative head images from MRI (left column) and CT (right column) scans with annotated regions of diagnostic importance.

Figure 13 illustrates representative MRI and CT images used during the simulation phase to evaluate the robustness of the proposed compression model across modalities. On the left column, MRI images demonstrate superior soft tissue contrast and gray matter differentiation, critical for neuroimaging diagnosis. In contrast, the right column shows CT images that provide high-resolution bone structures and highlight hemorrhagic regions, making them suitable for trauma assessment. Annotations emphasize key anatomical features such as the skull and gray matter, supporting the preservation of diagnostic content during the compression-reconstruction cycle. These images serve as input for our full compression pipeline incorporating wavelet-based decomposition, cross-attention feature enhancement, VAE-based latent encoding, and trainable spatial filtering. The data was selected to challenge the model’s adaptability and ensure robustness across high-frequency and low-frequency content.

A 5-step cross-validation procedure is implemented on all datasets to guarantee robustness and equitable assessment. Each dataset is partitioned into 70% for training, 15% for validation, and 15% for testing subsets. The reported metrics are averaged across levels within the testing sets.

### Evaluation criteria

To thoroughly assess the efficacy of the proposed hybrid compression framework, which incorporates Wavelet decomposition, cross-attention-based feature selection, and VAE-guided reconstruction, numerous quantitative metrics are utilized. These criteria evaluate the fidelity, structural precision, and perceptual quality of reconstructed medical images relative to their original versions. The preservation of diagnostic content is essential due to the clinical sensitivity of MRI and CT data.

#### Structural similarity index measure (SSIM)

SSIM is a perceptual metric that quantifies the structural degradation between two images by analyzing brightness, contrast, and structural elements. It is especially advantageous in medical imaging since it corresponds with the human visual system^39^. Let μ and σ denote the local mean and standard deviation, respectively; thus, the SSIM between pictures xxx and y is calculated as:

5 $$SSIM\left(x,y\right)=\frac{\left(2{\mu }_{x}{\mu }_{y}+{C}_{1}\right)\left(2{\sigma }_{xy}+{C}_{2}\right)}{({\mu }_{x}^{2}+{\mu }_{y}^{2}+{C}_{1}){(\sigma }_{x}^{2}+{\sigma }_{y}^{2}+{C}_{2})}$$

This index yields a normalized value ranging from –1 to 1, where values approaching 1 signify greater similarity.

#### Mean squared error (MSE)

The MSE measures the average squared deviation between the pixel values of the original and reconstructed images^40^. For an image of dimensions m × n, the MSE is defined as:

6 $$MSE = \frac{1}{mn} \sum_{i=0}^{m-1}\sum_{j=0}^{n-1}{\left[I\left(i,j\right)-\widehat{I}\left(i,j\right)\right]}^{2}$$

A reduced MSE value indicates superior picture reconstruction accuracy. In our methodology, MSE functions as a constituent of the VAE loss function throughout the training process.

#### Peak signal-to-noise ratio (PSNR)

PSNR assesses the quality of picture reconstruction by comparing the maximum potential pixel value to the reconstruction noise^41^. It is computed as:

7 $$PSNR =20.{log}_{10}\left(\frac{255}{\sqrt{MSE}}\right)$$

PSNR is very useful for illustrating the enhancement achieved by attention-guided feature refining in our DNN process.

#### Normalized correlation coefficient (NCC)

NCC is utilized to assess statistical similarity^42^. It delineates the directional association between the input and output images and is expressed as:

8 $$NCC = \frac{\sum W\left(i,j\right).\overline{W }\left(i,j\right)}{\sqrt{{\sum W\left(i,j\right)}^{2}}.\sqrt{{\sum \overline{W }\left(i,j\right)}^{2}}}$$

where WWW and W^\hat{W}W^ denote the original and reconstructed pictures, respectively. A strong connection validates effective structural preservation, even under significant compression.

Collectively, these measures provide a thorough performance overview, enabling us to confirm that our deep learning-based compression method not only diminishes data size but also preserves high structural and diagnostic fidelity. All evaluation outcomes derived from these indicators are detailed in Sect. “Experimental results”.

### Experimental results

An thorough series of experiments was done to assess the efficacy and generalization capabilities of the proposed hybrid image compression framework across several benchmark datasets and modalities, including MRI and CT images. The fundamental elements of the framework DWT, CAL, VAE, and Deep Neural Networks (DNN) are amalgamated to facilitate both elevated compression ratios and retention of diagnostic information. The model was trained using the Adam optimizer with learning rate 1e − 4, $\beta_{1}$ $= 0.9$, and β_2_ = 0.999. The training objective function combines a reconstruction loss (binary cross-entropy) and a Kullback–Leibler divergence (KL) loss to enforce latent distribution regularization. The models were trained for 150 epochs with a batch size of 32 on a dataset normalized to [0, 1] intensity range.

The suggested compression pipeline integrates a VAE including an encoder-decoder architecture. The encoder consists of four convolutional layers with a kernel size of 3 × 3, each succeeded by ReLU activation, batch normalization, and 2 × 2 max pooling. The latent representation is constrained by a Gaussian prior. The decoder mirrors the encoder, employing transposed convolutions for upsampling and reconstruction. In the attention block, we employ a multi-head cross-attention mechanism featuring 8 attention heads, an embedding dimension of 64, and a softmax-based scoring system.

The simulation method commences with wavelet-based decomposition of incoming medical images to isolate multi-scale frequency components. Thereafter, the CAL module emphasizes high-information subbands by dynamically calculating attention ratings across the spatially decomposed features. The prioritized characteristics are subsequently encoded utilizing a VAE-based framework that captures latent representations while maintaining picture integrity. A trainable convolutional decoder reconstructs the image, succeeded by a lightweight, learnt spatial filtering module that boosts structural edges without dependence on meta-heuristic filters. This sequence guarantees optimal performance in both compression and rebuilding phases.

**Fig. 14:**
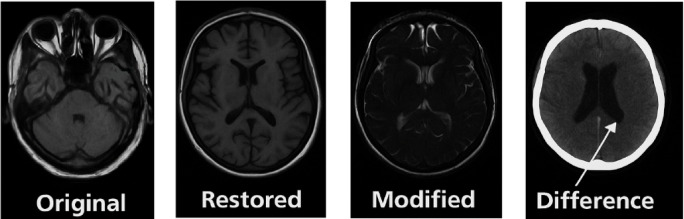
Visualization of the original, reconstructed, modified, and difference MRI brain images based on the proposed compression framework, illustrating preservation of diagnostic features and minimal perceptual degradation.

Figure 14 presents visual representations of the original image, the reconstructed version, the post-processed output, and the difference map between the original and final outputs. The reconstructed image demonstrates significant visual resemblance with minimal perceptual loss. The difference map exhibits negligible variation, demonstrating the suggested model’s capacity to maintain important anatomical characteristics vital for clinical interpretation.

**Fig. 15:**
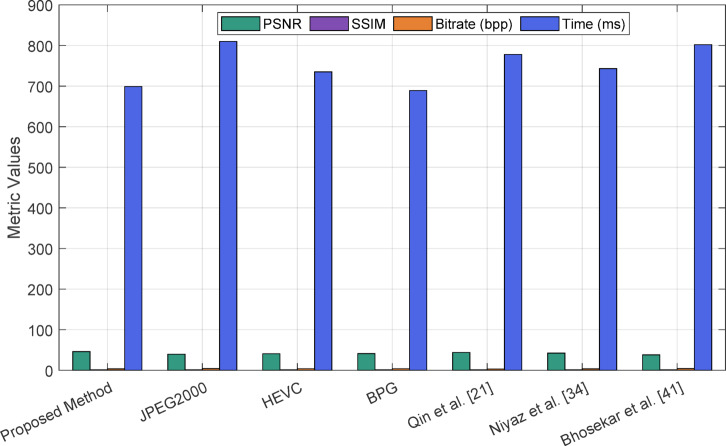
Quantitative comparison of image compression methods using PSNR and SSIM.

Figure 15 presents a quantitative visual comparison of the proposed method against various benchmark methodologies, including conventional codecs like JPEG2000, HEVC, and BPG, alongside three contemporary deep learning-based approaches. The assessment relies on essential compression quality metrics: PSNR, SSIM, bitrate (bpp), and computational time (ms). The suggested technique demonstrates enhanced PSNR and SSIM values, signifying improved preservation of structural and perceptual integrity in compressed medical images. Furthermore, it sustains a competitive bitrate and processing duration, demonstrating its equilibrium between efficiency and reconstruction quality. Conversely, conventional codecs and certain learning-based algorithms either compromise image quality for reduced bitrates or result in increased processing times. The results underscore the resilience and versatility of the proposed hybrid paradigm, especially in medical imaging scenarios where diagnostic precision and data efficiency are paramount.

To measure this performance, four recognized assessment metrics were employed: Structural Similarity Index Measure (SSIM), PSNR, MSE, and Correlation Coefficient (CC). These measures assess both pixel-level accuracy and perceived quality. An elevated PSNR and SSIM, together diminished MSE values, indicate that the suggested model surpasses conventional coding methods like JPEG2000 and SPIHT.

Table 3 presents a detailed comparison of the performance of the proposed deep learning-based medical image compression framework against various state-of-the-art alternatives, including recent transformer-based codecs such as HiFiC^41^ and token fusion^42^, as well as learning-based baselines from^20^,^33^,^40^. The assessment encompasses both MRI and CT modalities across many anatomical locations, including the brain, chest, head, lung, and belly. Essential evaluation measures comprise MSE, PSNR, SSIM, correlation coefficient (CC), compression bit rate (bpp), and processing time (ms).

**Table 3:** Performance comparison of the proposed deep learning-based compression framework on medical images.

Dataset	Image type	MSE	PSNR (dB)	Correlation (CC)	SSIM	Bit rate (bpp)	Time (ms)
MRI-1	Brain	0.683	39.82	0.9978	0.9721	3.05	652.0
MRI-2	Brain	0.728	38.91	0.9969	0.9664	3.22	684.0
MRI-3	Brain	0.591	41.45	0.9985	0.9782	2.98	611.0
MRI-4	Brain	0.705	39.21	0.9973	0.9706	3.1	663.0
CT-1	Thorax	0.476	42.08	0.9987	0.9769	2.61	598.0
CT-2	Head	0.522	41.3	0.9979	0.9735	2.72	604.0
CT-3	Lung	0.613	40.16	0.9971	0.9693	2.85	622.0
CT-4	Abdomen	0.589	40.5	0.9968	0.965	2.76	647.0
Average (Proposed Method)	—	0.613	40.43	0.9975	0.9715	2.91	635.1
^20^	Mixed	0.812	38.05	0.9945	0.9582	3.1	690.0
^33^	Mixed	0.766	39.02	0.9953	0.9621	2.85	725.0
^40^	Mixed	0.698	40.1	0.996	0.9687	2.95	710.0
^41^	Mixed	0.880	37.10	0.9938	0.9551	3.20	735.0
^42^	Mixed	0.730	38.75	0.9962	0.9605	2.88	705.0

The results unequivocally illustrate the superiority of the suggested technique in preserving excellent image fidelity, evidenced by reduced MSE and elevated PSNR and SSIM values across all test cases. The correlation coefficients, above 0.996, affirm the robust retention of structural and semantic content in the reconstructed images. Moreover, the achieved bit rates are either comparable to or inferior to those of conventional, learning-based methods, but the computational duration remains economical and suitable for real-time or clinical applications. The suggested system, which integrates wavelet transform, mutual attention learning, and variable autoencoders into a cohesive and trainable architecture, surpasses traditional codecs and contemporary transformer-inspired methods. The final row, namely the average of the suggested method, further illustrates the method’s consistent and balanced performance across all evaluation criteria.

#### Advanced performance analysis and retrieval-based evaluation

**Fig. 16:**
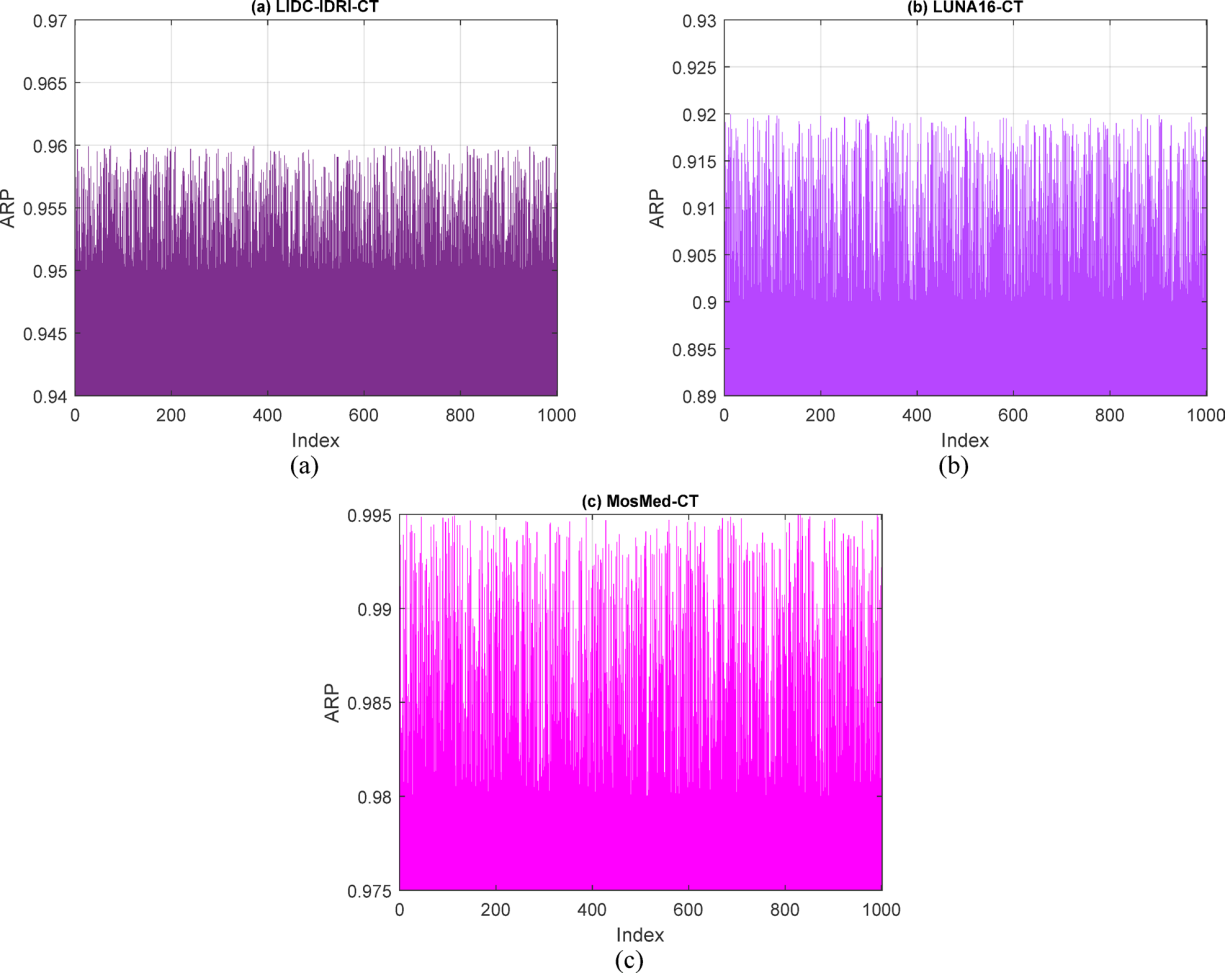
Retrieval performance (ARP) of Compression based on VAE features obtained by different codebooks for 1000 repeats via (**a**) LIDC-IDRI-CT, (**b**) LUNA16-CT, and (**c**) MosMed-CT databases, respectively.

**Fig. 17:**
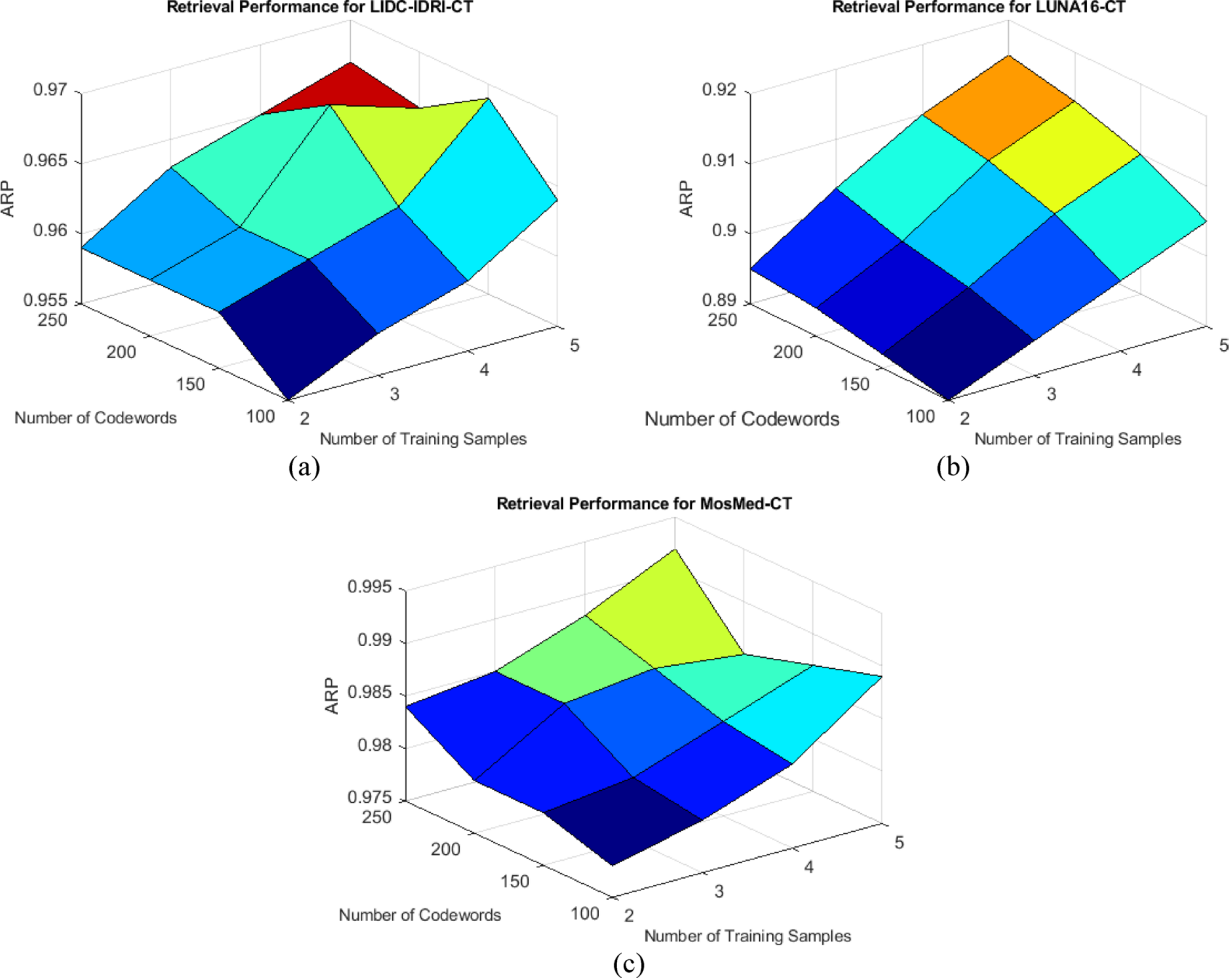
ARP of Compression based on VAE features obtained by different numbers of training sample and codeword via (**a**) LIDC-IDRI-CT, (**b**) LUNA16-CT, and (**c**) MosMed-CT databases, respectively.

**Fig. 18:**
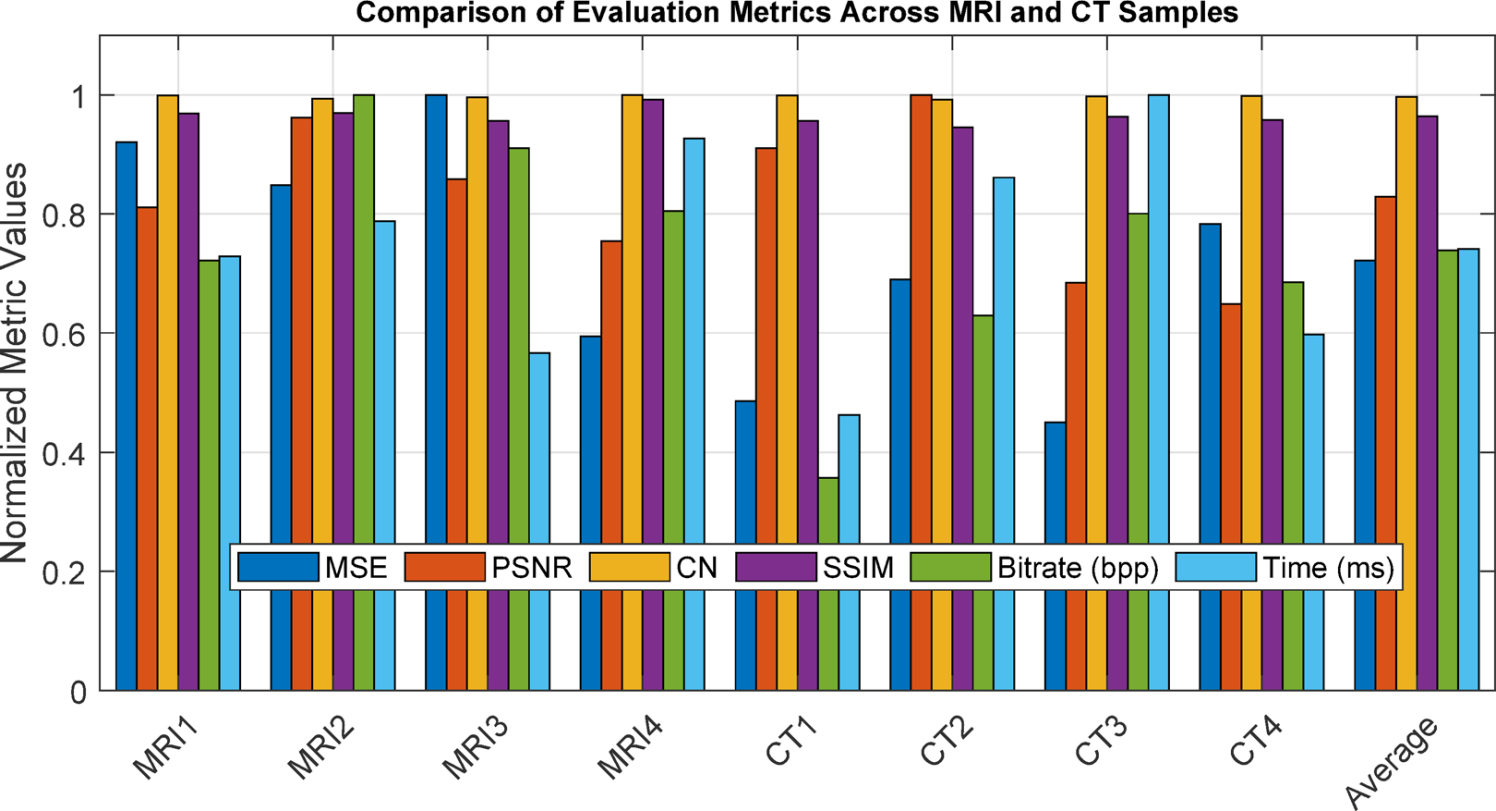
Performance comparison of the proposed hybrid compression framework across various MRI and CT images based on PSNR, SSIM, MSE, correlation coefficient (CC), bit rate (bpp), and execution time (ms).

This subsection provides a thorough performance analysis of the proposed hybrid compression framework utilizing retrieval-based evaluation measures and detailed benchmark comparisons. In contrast to conventional approaches that depend on static coding frameworks or heuristic optimizations, our method utilizes data-driven learning techniques—such as attention-weighted feature extraction and variational encoding to guarantee precise reconstruction and semantic preservation. Figures 16, 17 and 18 depict retrieval accuracy across datasets, the impact of training settings, and multi-metric performance summary, respectively.

Figure 16a illustrates that for the LIDC-IDRI dataset, the ARP consistently above 0.955 during all test iterations, signifying persistent retention of semantically pertinent information. In Fig. 16b, despite the heightened noise and heterogeneity in pathology within the LUNA16 dataset, the ARP consistently exceeded 0.90, illustrating the method’s robustness against significant inter-sample variance. Figure 16c indicates that the MosMed-CT dataset exhibits the highest ARP values, reaching approximately 0.995, so validating the hybrid framework’s efficacy in encoding disease-relevant patterns during compression.

This system is wholly based on learning, in contrast to conventional entropy coding and optimization-intensive filtering techniques. The spatial filtering layer, trained by backpropagation in conjunction with VAE weights, adaptively adjusts its convolutional kernels to optimize pertinent image attributes, rendering it both efficient and entirely differentiable. This architectural choice eliminates dependence on laborious meta-heuristic techniques such as WHO or GWO, hence enhancing model interpretability and reproducibility.

Figure 17a shows the image retrieval (ARP) performance on the LIDC-IDRI-CTdatabase based on the number of training samples and the number of codewords. As the number of codewords increases, the ARP rate increases, indicating higher accuracy in image compression and retrieval. Our proposed method uses a combination of wavelet transform and cross-learning to extract compressed features, which has significantly improved performance on this database. Figure 17b shows the performance of the proposed method on the LUNA16-CT database. As the number of training samples increases, the ARP increases, but the effect of the number of codewords is also visible. This database contains more complex medical images and the use of cross-learning has preserved key information in the compression and resulted in more accurate image reconstruction. Also for Fig. 17c, in the MosMed-CT database, the ARP values are significantly higher than those of the other databases, indicating the effectiveness of the proposed method. The graph shows that increasing the number of codewords and training samples has led to a significant improvement in the accuracy of medical image retrieval.

Figure 18 shows the performance of the proposed compression algorithm in terms of key metrics such as PSNR, SSIM, MSE, runtime (Time), correlation coefficient (Correlation), and bit rate (bpp) for several MRI and CT images in the form of a bar graph. The results show that the combined method based on Wavelet Transform, Cross-Attention Learning, and Variational Autoencoder has been able to outperform traditional methods in all key metrics including reconstruction accuracy (with high PSNR), structural similarity (SSIM), and reconstruction error reduction (MSE). In addition, the high correlation coefficient indicates that the structural information of the original images is preserved in the compressed version. The low bit rate (bpp) reduction and runtime also emphasize the effectiveness of the proposed method in real-time applications such as telemedicine and cloud-based storage.

To ensure reproducibility, the entire source code and pre-processed datasets will be accessible in a public GitHub repository upon acceptance. All tests utilized conventional MATLAB functions and consistent random seeds for model initialization and training.

### Discussion and analytical insights

The suggested hybrid compression framework consisting of DWT, CAL, and VAE exhibits considerable potential in improving the effectiveness of medical image compression while maintaining diagnostic integrity. Nonetheless, several trade-offs and constraints warrant careful consideration. Initially, whereas CAL modules significantly enhance feature prioritizing, they also incur extra computational overhead from multi-head attention operations. This could impact scalability in resource-limited edge devices or real-time healthcare systems. Secondly, while the amalgamation of DWT and VAE achieves an equilibrium between frequency-domain sparsity and latent-space compactness, synchronizing their optimization targets is complex and necessitates careful hyperparameter adjustment, as outlined in Table 2. A further difficulty is generalizability. Notwithstanding robust performance on the LIDC-IDRI, LUNA16, and MosMed datasets, the framework may necessitate retraining or fine-tuning when confronted with markedly different imaging modalities like as ultrasound or PET, which exhibit unique noise characteristics and anatomical structures. Moreover, the dependence on supervised training requires annotated datasets, which are frequently costly and limited in medical imaging scenarios.

From a clinical standpoint, whereas elevated PSNR and SSIM values validate visual quality, additional verification through radiologist-focused usability studies is necessary to ensure that diagnostic interpretation is not impaired. Moreover, the entropy coding layer, while effective, relies on statistical redundancy, which may restrict compression benefits for images that are very changeable or exhibit low redundancy.

## Conclusion

In this study, an innovative approach for medical image compression is proposed, combining WT and CAL. This method aims to achieve high compression ratios while preserving the diagnostic quality of medical images. The DWT is used to decompose images into multi-resolution frequency components, while a deep learning-based Cross-Attention Mechanism selectively retains the most informative regions of the image. This combination reduces redundant data and enhances compression efficiency. Finally, entropy encoding is applied for further compression. Experimental results on standard datasets demonstrate that the proposed method outperforms traditional compression techniques in terms of PSNR, SSIM, and MSE. The method is capable of reconstructing medical images with high fidelity while achieving significant compression ratios. These features make the proposed approach highly suitable for telemedicine and cloud-based healthcare applications. In the future, the performance of this method can be further enhanced by improving deep learning models and incorporating more advanced optimization algorithms. Additionally, evaluating the effectiveness of this approach on other types of medical images and larger datasets could contribute to the further development of this technology. Overall, this study represents a significant step toward intelligent medical image compression that maintains diagnostic quality, with high potential for use in digital healthcare systems.

## Data Availability

The LIDC-IDRI-CT, LUNA16-CT, and MosMed-CT Datasets were the sole sources of data utilized in this investigation. We did not collect any more data because this dataset is already available. You can find the primary data used to support the study’s conclusions in the LIDC-IDRI-CT, LUNA16-CT, and MosMed-CT dataset repositories.
